# Clinical characteristics and prognosis of patients with isolated thrombotic vs. obstetric antiphospholipid syndrome: a prospective cohort study

**DOI:** 10.1186/s13075-021-02515-w

**Published:** 2021-05-08

**Authors:** Hui Jiang, Chu-Han Wang, Nan Jiang, Jing Li, Chan-Yuan Wu, Qian Wang, Meng-Tao Li, Xin-Ping Tian, Jiu-Liang Zhao, Yan Zhao, Xiao-Feng Zeng

**Affiliations:** 1 Department of Rheumatology and Clinical Immunology, Chinese Academy of Medical Sciences & Peking Union Medical College, Beijing, China; 2 National Clinical Research Center for Dermatologic and Immunologic Diseases, Ministry of Science & Technology, Beijing, China; 3 Key Laboratory of Rheumatology and Clinical Immunology, Ministry of Education, Beijing, China; 4 State Key Laboratory of Complex Severe and Rare Diseases, Peking Union Medical College Hospital, Chinese Academy of Medical Sciences & Peking Union Medical College, Beijing, China

**Keywords:** Antiphospholipid syndrome, Thrombotic, Recurrence, Obstetric, Phenotype

## Abstract

**Background:**

Several studies suggested that thrombotic and obstetric antiphospholipid syndromes could be independent identities, but few have systematically compared their clinical characteristics and prognosis.

**Objective:**

The objective of this study is to identify key differences between thrombotic APS (tAPS) and obstetric APS (oAPS).

**Methods:**

This single-center, prospective study included consecutive patients with primary antiphospholipid syndrome (APS) receiving treatment at the Peking Union Medical College Hospital during a period from 2013 to 2020.

**Results:**

Screening of the database yielded a total of 244 women with positive antiphospholipid antibody (aPL). Among the 105 women with primary APS, 39 (37.14%) had isolated tAPS (ItAPS), 44 (41.90%) had isolated oAPS (IoAPS), and 9 (8.57%) had both tAPS and tAPS+oAPS. In comparison to those with IoAPS, patients with ItAPS had older age (41.92 ± 11.97 vs. 33.16 ± 4.22 years, *P* < 0.01), higher rate of cardiovascular risk (at least one positive of coronary heart disease, hypertension, obesity, diabetes, and hyperlipidemia) (41.03% vs. 6.82%, *P* < 0.01), and higher frequency of thrombocytopenia (43.59% vs. 20.45%, *P* < 0.05). Antibody profiles were generally similar among the groups, but isolated anti-β2GPI positivity was more common in patients with IoAPS (52.27% vs. 17.94% for ItAPS, *P* = 0.01). Triple aPL positivity was more common in patients with both tAPS and oAPS (66.67% vs. 46.15% for ItAPS vs. 25% for IoAPS, *P* = 0.022). Blood homocysteine was higher in patients with ItAPS (11.20 vs. 9.90 μmol/L for IoAPS, *P* < 0.05), but there were no differences in inflammatory markers or complements. Recurrence rate of thrombosis was higher in patients with ItAPS (33.33% vs. 2.27% for IoAPS, *P* ≤ 0.001) with a mean follow-up of 61 months.

**Conclusion:**

Despite generally similar antibody and biochemical profiles, patients with ItAPS had much higher risk of recurrent thrombosis than IoAPS, supporting distinct mechanisms of pathogenesis.

## Introduction

Based on the 2006 revised Sydney criteria, antiphospholipid syndrome (APS) is defined as prolonged positive of antiphospholipid antibody (aPL) with thrombotic and/or obstetric manifestations. Traditional aPLs include anticardiolipin antibody (ACL), anti-β2 glycoprotein antibody (anti-β2GPI), and lupus anticoagulant (LA) [1, 2]. Thrombotic APS (tAPS) and obstetric APS (oAPS) share similar antibody profiles and manifestations, but may represent distinct diseases [3–5]. Specifically, antigen distribution and inflammatory status differ between the two variants [6], but comparative data on clinical features and prognosis are limited. We conducted a longitudinal study to compare clinical characteristics and antibody profiles in patients with isolated tAPS (ItAPS) vs. isolated oAPS (IoAPS). The adjusted Global Anti-Phospholipid Syndrome Score (aGAPSS) conceived by Savino Sciascia et al. was used to assess thrombotic risk, combing cardiovascular risks and aPL positivity [7, 8].

## Materials and methods

### Patients and data

This is a single-center, prospective cohort study. The study was based on a database at the National Clinical Research Center for Dermatologic and Immunologic Diseases (NCRC-DID) at the Peking Union Medical College Hospital and included 244 patients with prolonged positive aPL between 2013 and 2020. The NCRC-DID recorded clinical characteristics, biochemical analyses, antibody profiles, and thrombotic events every 6 months. Only women with primary APS were included in the current study. ItAPS was defined as prolonged positive aPL plus a history of thrombosis but with no characteristic obstetric complications at the time of diagnosis. IoAPS was defined as prolonged positive aPL with characteristic obstetric complications but no thrombosis history at the time of diagnosis. Patients with both tAPS and oAPS (tAPS+oAPS) at the time of diagnosis were analyzed as a separate group.

### Statistical methods

Continuous variables are expressed as the mean and standard deviation (SD) or the median and quartile (Q1, Q3). Comparison between ItAPS and IoAPS was conducted using Student’s *t* test or Wilcoxon-Mann-Whitney test. The Kruskal-Wallis *H* test was used to compare the three groups. Categorical variables are expressed as the number and percentage and analyzed using Fisher’s exact test or chi-square test as appropriate. A log-rank test was used to compare thrombotic recurrence during follow-up. Time to recurrence was defined as the interval between diagnosis and first recurrence of thrombosis and compared using the Kaplan-Meier method. Cox’s proportional hazards regression model was used to assess the risk factors of recurrent thromboses. *P* < 0.05 (2-sided) was considered statistically significant.

## Results

### Demographics and baseline laboratory results

**Fig. 1:**
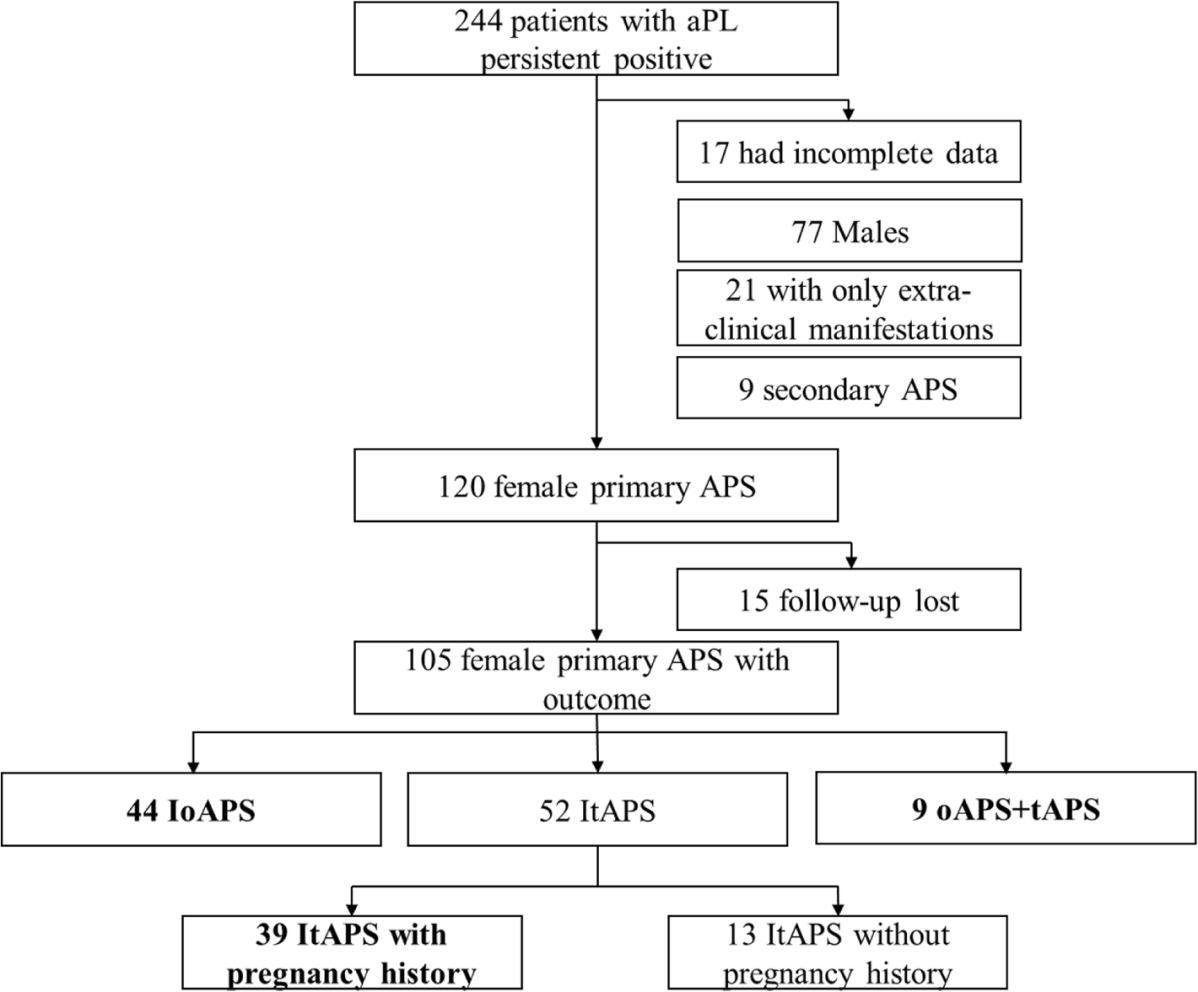
APS cohort in the Peking Union Medical College Hospital database. A total of 244 patients with persistent positive of aPL were followed up, including those with primary APS, secondary APS, and non-criteria APS. After excluding 17 patients with incomplete data, 77 males, 21 patients with only extra-clinical manifestations, 9 female secondary APS patients, and 15 female primary APS lost to follow-up, a total of 105 female primary APS patients with outcome were enrolled in the cohort. Our study included 39 patients with ItAPS and pregnancy history, 44 patients with IoAPS, and 9 patients with tAPS+oAPS. Patients who lacked a history of pregnancy were excluded

The database included a total of 244 patients with persistent positive aPL. One hundred thirty-nine were excluded from the final analysis: 17 with incomplete data, 77 male patients, 21 women with non-criteria APS (NC-APS), and 9 women with secondary APS. Of the remaining 120 women with primary APS, 15 patients who were lost to follow-up were also excluded. The final analysis included a total of 105 women with primary APS. Among these patients, 13 (12.38%) had ItAPS but no history of pregnancy (these patients were not enrolled in statistical analysis), 39 (37.14%) had ItAPS (median duration of 51 months) and pregnancy histories, 44 (41.90%) had IoAPS (median duration of 48.5 months), and 9 (8.57%) had both tAPS and oAPS (median duration of 41 months) (Fig. 1).

In comparison to patients with IoAPS, patients with ItAPS were older (41.92 ± 11.97 vs. 33.16 ± 4.22 years for IoAPS, *P* < 0.001) and had higher body mass index (24.60 ± 4.20 vs. 22.84 ± 3.21 kg/m^2^ for IoAPS, *P* < 0.05) at baseline (Table 1). Sixteen (41.03%) patients with ItAPS had at least one cardiovascular risk factor, whereas only 3 (6.82%) patients with IoAPS and 3 (33.33%) patients with tAPS+oAPS had at least one cardiovascular risk factor (ItAPS vs. IoAPS vs. tAPS+oAPS, *P* = 0.002). No differences were found in the histories of smoking, coronary heart disease, diabetes mellitus, and hyperlipidemia among the three groups. In terms of non-criteria APS manifestations, patients with ItAPS tended to experience thrombocytopenia more frequently compared to IoAPS (43.59% vs. 20.45%, *P* = 0.033). Patients with tAPS+oAPS had significantly higher aGAPSS (combined with aPL, hyperlipidemia, and hypertension) vs. other groups (*P* = 0.001). Complications did not differ among the three groups.

**Table 1:** Demographic characteristics

	IoAPS (*n* = 44)	ItAPS (*n* = 39)	tAPS+oAPS (*n* = 9)	*P* value All groups	*P* value IoAPS vs. ItAPS
Age (years), mean ± SD	33.16 ± 4.22	41.92 ± 11.97	35.22 ± 3.82	**0.000**	**0.000**
BMI (kg/m^2^), mean ± SD	22.84 ± 3.21	24.60 ± 4.20	24.61 ± 2.69	0.086	**0.024**
Duration, months median (Q1, Q3)	48.50 (36.00, 77.00)	51.00 (22.00, 93.00)	41.00 (15.00, 61.00)	0.657	0.404
Smoking history, *n* (%)	1 (2.27)	1 (2.56)	0	1.000	1.000
Cardiovascular risk factors, *n* (%)	3 (6.82)	16 (41.03)	3 (33.33)	**0.002**	**0.000**
Coronary heart disease	0 (0)	3 (7.69)	0	0.219	0.099
Hypertension	1 (2.27)	6 (15.38)	2 (22.22)	**0.028**	**0.048**
Obesity (BMI > 30 kg/m^2^)	2 (4.55)	8 (20.51)	0	**0.048**	**0.040**
Diabetes mellitus	0 (0)	0 (0)	0	–	–
Hyperlipidemia	0 (0)	3 (7.69)	1 (11.11)	0.107	0.099
Complications, *n* (%)	12 (27.27)	20 (51.28)	4 (44.44)	0.079	**0**.**041**
Kidney disease	2 (4.55)	3 (7.69)	0	0.799	0.662
Thrombocytopenia	9 (20.45)	17 (43.59)	4 (44.44)	0.056	**0.033**
Non-stroke CNS manifestations	1 (2.27)	3 (7.69)	0	0.573	0.337
Valvular heart disease	4 (9.09)	3 (7.69)	1 (11.11)	1.000	1.000
aGAPSS median (Q1, Q3)	4 (4, 12)	10 (4, 13)	13 (6, 15)	**0.001**	**0.001**

*BMI*, body mass index; *cardiovascular risk factors and complications positive*, patients with at least one positive subgroup symptom; *CNS*, central nervous system; *aGAPSS*, adjusted Global Anti-Phospholipid Syndrome Score

Isolated anti-β2GPI positivity (negative ALC and LA) was more common in the IoAPS group (52.27% vs. 17.94% for tAPS and 11.11% for tAPS+oAPS, *P* = 0.001). Triple aPL positivity was more common in patients with tAPS+oAPS (66.67% vs. 46.15% for ItAPS and 25.00% for IoAPS, *P* = 0.022). In comparison to the IoAPS group, patients with ItAPS had higher blood homocysteine [11.20 (9.70, 14.60) vs. 9.9 (8.10, 12.10) μmol/L, *P* < 0.05], but similar biochemical and antibody profiles otherwise (Table 2).

**Table 2:** Laboratory test results

	IoAPS (*n* = 44)	ItAPS (*n* = 39)	tAPS+oAPS (*n* = 9)	*P* value All groups	*P* value IoAPS vs. ItAPS
Antibody categories					
Triple positive, *n* (%)	11 (25.00)	18 (46.15)	6 (66.67)	**0.022**	0.065
Double positive, *n* (%)	7 (15.90)	9 (23.08)	1 (11.11)	0.678	0.578
ACL+LA	0 (0)	1 (2.56)	0 (0)	0.521	0.470
LA+anti-β2GPI	2 (4.55)	2 (5.13)	1 (11.11)	0.632	1.000
ACL+anti-β2GPI	5 (11.36)	6 (15.38)	0 (0)	0.651	0.748
Single positive, *n* (%)	26 (59.10)	12 (30.77)	2 (22.22)	**0.017**	**0.015**
ACL	1 (2.27)	1 (2.56)	0 (0)	1.000	1.000
Anti-β2GPI	23 (52.27)	7 (17.94)	1 (11.11)	**0.001**	**0.001**
LA	2 (4.55)	4 (10.26)	1 (11.11)	0.420	0.413
ESR (mm/h), median (quartile)	10 (6, 27)	10 (5,21)	11 (4.5,27.5)	0.814	0.254
CRP (mg/L), median (quartile)	1.03 (0.56, 3.85)	1.32 (0.50,3.92)	9.55 (0.46,10.95)	0.808	0.383
Hcy (μmol/L), median (quartile)	9.90 (8.10, 12.10)	11.20 (9.70,14.60)	10.85 (9.08,12.68)	0.152	**0.028**
C3 (g/L), median (quartile)	0.98 (0.81, 1.17)	0.92 (0.73,1.12)	0.75 (0.66,1.11)	0.235	0.129
C4 (g/L), median (quartile)	0.16 (0.13, 0.23)	0.17 (0.13,0.21)	0.14 (0.12,0.18)	0.565	0.371

*ACL*, anticardiolipin antibody; *anti-β2GPI*, anti-β2 glycoprotein I antibody; *LA*, lupus anticoagulant; *ESR*, erythrocyte sedimentation rate; *CRP*, C-reactive protein; *Hcy*, homocysteine; *C3*, complement C3; *C4*, complement C4

### Thrombotic events at diagnosis and follow-up between ItAPS and IoAPS

At the baseline, patients had 68 thrombotic events, all in patients with ItAPS. Twenty-five patients (accounting for 64.10% in patients with ItAPS) presented with arterial thrombotic events, and 23 (58.97%) presented with venous thrombotic events, including 7 pulmonary embolisms, 3 myocardial infarctions, and 8 strokes (Table 3). Sixteen patients (41.03%) had recurrent thrombosis prior to the time of diagnosis.

**Table 3:** Thrombotic events at diagnosis and follow-up

	Before diagnosis	Follow-up			
Thromboses (*n*)	IoAPS (*n* = 44)	ItAPS (*n* = 39)	IoAPS (*n* = 44)	ItAPS (*n* = 39)	*P* value
Total thromboses, *n* (%)	0	39 (100.00)	1 (2.27)	13 (33.33)	**0.000**
Arterial thrombosis, *n* (%)	0	25 (64.10)	0 (0.00)	9 (23.08)	**0.001**
Venous thrombosis, *n* (%)	0	23 (58.97)	1 (2.27)	5 (10.26)	0.094
Pulmonary embolism, *n* (%)	0	7 (17.95)	0 (0.00)	5 (10.26)	**0.020**
Myocardial infarction, *n* (%)	0	3 (7.69)	0 (0.00)	0 (0.00)	
Stroke, *n* (%)	0	8 (20.51)	0 (0.00)	1 (5.13)	0.470
Recurrent thromboses, *n* (%)	0	16 (41.03)	0 (0.00)	5 (10.26)	**0.020**

With the average follow-up of 61 ± 43 months, 13 (33.33%) patients with ItAPS experienced new thrombotic events, 9 patients (23.08%) had arterial thromboses, and 5 patients (10.26%) had venous thromboses. Only 1 patient (2.27%) with IoAPS experienced a new thrombotic event (*P* < 0.001 vs. ItAPS). A total of 5 patients (10.26%), all in the ItAPS group, experienced recurrent thrombotic events during the follow-up.

### Survival analysis

**Fig. 2:**
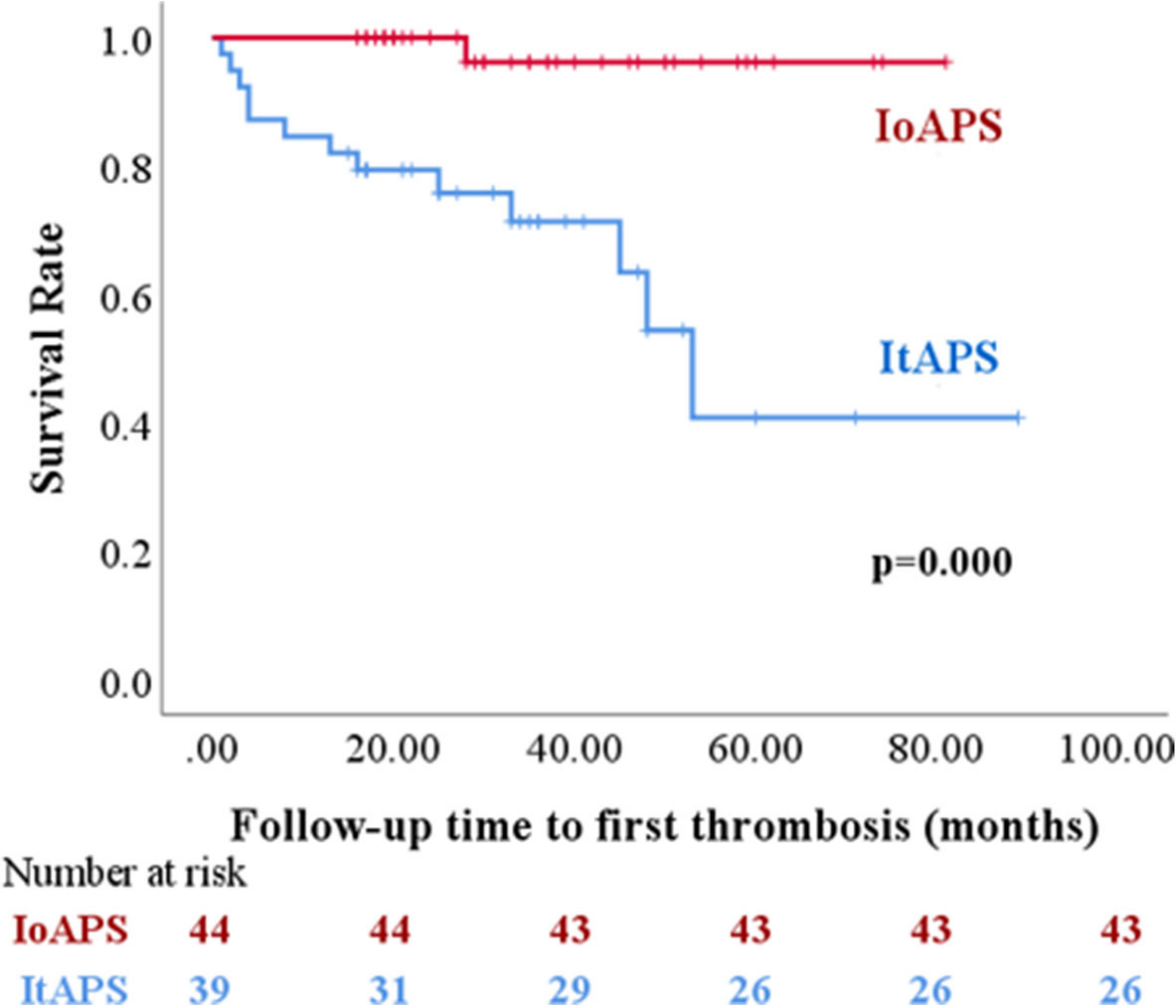
Kaplan-Meier survival curves after the first thrombosis. The curve shows the time to first thrombotic recurrence during follow-up in patients with IoAPS and ItAPS

Kaplan-Meier analyses indicated a shorter time to first thrombotic recurrence in patients with ItAPS vs. IoAPS (*P* < 0.001; Fig. 2). In the univariate Cox regression, risk of recurrent thromboses included cardiovascular events [hazard ratio (HR) 3.316, 95% confidence interval (CI) 1.140–9.646, *P* = 0.028] and thromboses (HR 17.115, 95% CI 2.237–130.927, *P* = 0.006) (Table 4). In a multivariate Cox regression that adjusted for age, a history of isolated thrombosis was an independent risk for recurrent thromboses (HR 15.791, 95% CI 1.747–142.763, *P* = 0.014).

**Table 4:** Univariate and multivariate Cox regression of predictors of recurrent thromboses in patients with APS

Variables	Univariate regression			Multivariate regression		
	HR	95% CI	*P* value	HR	95% CI	*P* value
ItAPS phenotype	17.115	2.237–130.927	**0.006**	15.791	1.747–142.763	**0.014**
Age (per 10 years)	0.857	0.417–1.762	0.674			
High homocysteine level^a^	1.013	0.223–4.597	0.987			
Cardiovascular risk	3.316	1.140–9.646	**0.028**			
Triple positive antibodies	1.433	0.494–4.155	0.508			
Single anti-β2GPI positive	0.382	0.084–1.730	0.212			
Thrombocytopenia	2.030	0.079–5.814	0.187			

^a^High homocysteine level is defined as homocysteine > 15 μmol/L

*HR*, hazard ratio; *CI*, confidence interval

## Discussion

The results from the current study demonstrated several differences between primary ItAPS and IoAPS, including age, BMI, baseline cardiovascular disease, and thrombocytopenia in demographics. Nearly one-half of the patients with ItAPS had at least one cardiovascular risk factor vs. only 6.82% in the patients with IoAPS. This is consistent with several previous studies that associated arterial thrombotic events in patients with APS with traditional cardiovascular risk factors, including hypertension, diabetes, hypercholesterolemia, and smoking [9–14]. In the current study, the rate of thrombocytopenia was higher in patients with ItAPS vs. IoAPS. Such a finding is consistent with a previous study by Hisada et al., in which low platelet count was associated with increased risk of thrombotic events in aPL carriers [15].

Avivi et al. reported that 50% of patients with APS and hyperhomocysteinemia had thrombotic events [16], and Kassis et al. reported an increased risk of arterial thrombosis in patients with aPL and high homocysteine levels [17]. In the current study, the multivariate regression failed to identify increased homocysteine as a risk for recurrent thrombotic events, but subjects with ItAPS had higher blood homocysteine than IoAPS. More studies are needed to verify the potential link. Anti-β2GPI overexpression was associated with a high incidence of obstetric complications in previous studies [18, 19], while they did not compare single anti-β2GPI positivity between ItAPS and IoAPS. In the current study, the percentage of the patients with isolated anti-β2GPI positivity (negative ACL and LA) was much higher in the IoAPS group vs. in the ItAPS. The current study also found a high rate of triple aPL positivity in patients with tAPS+oAPS (66.67%) compared to ItAPS (46.15%) and IoAPS (25%).

This is consistent with previous studies, in which triple aPL positivity could be a risk factor of thrombotic events [20]. In our opinion, high aGAPSS in patients with tAPS+oAPS could be partly attributed to the high rate of triple aPL positivity compared to tAPS.

Recurrent thrombosis occurred in approximately one-third of patients with ItAPS in this study, and up to 10.26% of patients with ItAPS had more than one recurrence. This is consistent with studies that recurrence thrombosis is common in patients with tAPS and rare in patients with oAPS [3, 21, 22]. Bazzan et al. reported that 31% of patients with tAPS experienced recurrent thromboses during the first 5 years after diagnosis (with or without a pregnancy, including primary APS and secondary APS) [23], and a similar study reported around 52% recurrence rate in patients with thrombotic histories (with or without a pregnancy, primary APS, including male and female), but only a 19% recurrence rate in patients with only oAPS during 18 years of follow-up [4]. Several studies reported a high risk of thrombosis in patients with oAPS [24, 25], but these studies lacked a straightforward comparison to patients with ItAPS. Although cardiovascular risks, thrombocytopenia, hyperhomocysteinemia, anti-β2GPI, and triple aPL positivity presented a difference between ItAPS and IoAPS, the multivariate analysis failed to show an association with recurrent thrombotic events.

The mechanisms behind the distinctive prognosis in ItAPS vs. IoAPS are unknown. In the “second hit” hypothesis, positive aPL represents a prethrombotic state, and a second hit of thrombophilic conditions, such as infection, inflammation, and trauma, are needed to trigger a thrombotic event [6, 26]. A pedigree study showed that thrombosis rarely occurs without a “second hit” [27]. Both in vivo and in vitro experiments showed that anti-β2GPI only binds to non-resting vascular endothelial cells (ECs), and thrombosis only forms in the presence of proinflammatory factors [28, 29]. Patients with oAPS may not require a “second hit” due to the overexpression of β2GPI on decidual ECs and trophoblasts [28, 30, 31]. The hormonal changes in patients with oAPS may serve as de facto “second hit” and cause placental dysfunction and abnormal vascular changes during pregnancy [6, 32, 33]. aPL may lead to different manifestations through distinct mechanisms. Lambrianides et al. reported that only aPL from patients with vascular thrombosis but no obstetric morbidity stimulates the phosphorylation of nuclear factor kappa-light-chain-enhancer of activated B cells (NFκB) and p38 mitogen-activated protein kinase and increases tissue factor activity in monocytes, thus associated with hypercoagulability in APS [34]. In vitro study by Poulton et al. showed that only aPL from patients with obstetric morbidity but no vascular thrombosis could inhibit trophoblast invasion [35]. Together, these findings may explain the higher complication rate in patients with ItAPS vs. IoAPS and the higher positive anti-β2GPI rate of IoAPS in our cohort. Therefore, tAPS could be categorized as an independent phenotype from oAPS both clinically and pathologically. A major strength in the current study is the exclusion of secondary APS from data analysis. Limitations include insufficient sample size and follow-up time and thus power to fully identify the differences and risk factors.

## Conclusions

Differences in baseline characteristics and recurrence observed in the current study suggest ItAPS and IoAPS represent independent forms of APS.

## Data Availability

Data are available from the corresponding authors upon request.

## Abbreviations

APS: Antiphospholipid syndrome
ItAPS: Isolated thrombotic antiphospholipid syndrome
IoAPS: Isolated obstetric antiphospholipid syndrome
aPLs: Antiphospholipid antibodies
anti-β2GPI: Anti-β2-glycoprotein antibody
tAPS: Thrombotic antiphospholipid syndrome
oAPS: Obstetric antiphospholipid syndrome
NCRC-DID: National Clinical Research Center for Dermatologic and Immunologic Diseases
